# Invaders, natives and their enemies: distribution patterns of amphipods and their microsporidian parasites in the Ruhr Metropolis, Germany

**DOI:** 10.1186/s13071-015-1036-6

**Published:** 2015-08-13

**Authors:** Daniel S. Grabner, Alexander M. Weigand, Florian Leese, Caroline Winking, Daniel Hering, Ralph Tollrian, Bernd Sures

**Affiliations:** Aquatic Ecology and Centre for Water and Environmental Research, University of Duisburg-Essen, Universitaetsstr. 5, 45141 Essen, Germany; Department of Animal Ecology, Evolution and Biodiversity, Ruhr-University Bochum, Universitaetsstr. 150, 44801 Bochum, Germany; Department of Zoology, University of Johannesburg, PO Box 524, Auckland Park 2006, Johannesburg, South Africa; Present address: Aquatic Ecosystems Research, University of Duisburg-Essen, Universitaetsstr. 5, 45141 Essen, Germany; Centre for Water and Environmental Research, University of Duisburg-Essen, Universitaetsstr. 5, 45141 Essen, Germany

**Keywords:** Invasive amphipods, DNA barcoding, Microspora, Parasite spillover, Spillback, Enemy release

## Abstract

**Background:**

The amphipod and microsporidian diversity in freshwaters of a heterogeneous urban region in Germany was assessed. Indigenous and non-indigenous host species provide an ideal framework to test general hypotheses on potentially new host-parasite interactions, parasite spillback and spillover in recently invaded urban freshwater communities.

**Methods:**

Amphipods were sampled in 17 smaller and larger streams belonging to catchments of the four major rivers in the Ruhr Metropolis (Emscher, Lippe, Ruhr, Rhine), including sites invaded and not invaded by non-indigenous amphipods. Species were identified morphologically (hosts only) and via DNA barcoding (hosts and parasites). Prevalence was obtained by newly designed parasite-specific PCR assays.

**Results:**

Three indigenous and five non-indigenous amphipod species were detected. *Gammarus pulex* was further distinguished into three clades (C, D and E) and *G. fossarum* more precisely identified as type B. Ten microsporidian lineages were detected, including two new isolates (designated as *Microsporidium* sp. nov. RR1 and RR2). All microsporidians occurred in at least two different host clades or species. Seven genetically distinct microsporidians were present in non-invaded populations, six of those were also found in invaded assemblages. Only *Cucumispora dikerogammari* and *Dictyocoela berillonum* can be unambiguously considered as non-indigenous co-introduced parasites. Both were rare and were not observed in indigenous hosts. Overall, microsporidian prevalence ranged from 50 % (in *G. roeselii* and *G. pulex* C) to 73 % (*G. fossarum*) in indigenous and from 10 % (*Dikerogammarus villosus*) to 100 % (*Echinogammarus trichiatus*) in non-indigenous amphipods. The most common microsporidians belonged to the *Dictyocoela duebenum-* /*D. muelleri-* complex, found in both indigenous and non-indigenous hosts. Some haplotype clades were inclusive for a certain host lineage.

**Conclusions:**

The Ruhr Metropolis harbours a high diversity of indigenous and non-indigenous amphipod and microsporidian species, and we found indications for an exchange of parasites between indigenous and non-indigenous hosts. No introduced microsporidians were found in indigenous hosts and prevalence of indigenous parasites in non-indigenous hosts was generally low. Therefore, no indication for parasite spillover or spillback was found. We conclude that non-indigenous microsporidians constitute only a minimal threat to the native amphipod fauna. However, this might change e.g. if *C. dikerogammari* adapts to indigenous amphipod species or if other hosts and parasites invade.

**Electronic supplementary material:**

The online version of this article (doi:10.1186/s13071-015-1036-6) contains supplementary material, which is available to authorized users.

## Background

Globalization of economy has fundamentally changed biodiversity patterns on our planet [1, 2]. Species and their parasites are frequently transported over thousands of kilometers into new habitats, encounter new organismal communities and may initiate a cascade of biological interactions [3–5]. In particular, urban areas are hotspots of species introductions [6, 7], yet simultaneously presenting an ideal platform to test ecological and evolutionary hypotheses on host-parasite interactions between indigenous and non-indigenous communities. With over five million people the Ruhr Metropolis in North Rhine-Westphalia (NRW) is the most densely populated region in Germany and a major entry point for non-indigenous species introduced from all over the world, in particular into freshwater ecosystems.

Streams and rivers in the region belong to the catchments of the rivers Lippe, Emscher and Ruhr, and flow into the Rhine, which also crosses the region. The Ruhr Metropolis offers a broad range of environmental conditions: streams remained in a near-natural state, while others were used as open sewage channels for about 100 years; some of the latter have been partly restored or even newly created in recent times. Due to this mosaic of ecological conditions, which can be considered as migration obstacles in several cases, not all stream sections are directly accessible for organisms spending their entire life cycle in the water. As an example, direct immigration of non-indigenous free-living species from the River Rhine to some of the Emscher tributaries is not possible, since the Emscher exclusively transports sewage and is hostile for most organisms except bacteria or some oligochaete species.

Amphipod crustaceans are particularly successful invaders of freshwater ecosystems; they are also among the first colonizers of newly available (urban) freshwater habitats. As ecological keystone species they show predatory, decomposing and scavenging feeding habits [8, 9]. For NRW, 14 amphipod species are known from epigean freshwater habitats [10]. Three species are indigenous (*Gammarus fossarum*, *G. pulex* and *G. roeselii*), while all others were introduced, for example from the Atlantic region of France (*Echinogammarus berilloni*), North America (e.g. *G. tigrinus*, *Crangonyx pseudogracilis*) or the Ponto-Caspian region (e.g. *Dikerogammarus villosus*, *D. haemobaphes*, *E. trichiatus*) [11, 12].

Amphipod assemblages of restored or newly built urban freshwater habitats in the Ruhr Metropolis frequently consist of a mixture of indigenous and non-indigenous species [13]. Introduced amphipods might not only directly influence native communities, e.g. by predation, competition and eventual replacement [14], but also indirectly through its co-introduced parasites (see [4]) like the microsporidian *Cucumispora dikerogammari* that might be transmitted from *D. villosus* to indigenous host species (parasite spillover [15–19]). At the same time, the new arrivers are exposed to indigenous parasites leading to new host-parasite associations, potential parasite spillback [20] and an increased ecological complexity of the invaded freshwater communities [5, 16, 21, 22].

Microsporidians are among the most common parasites of amphipods; they are a group of highly reduced (e.g. lack of mitochondria, small ribosomes) unicellular fungi present in vertebrate and invertebrate species [23–25]. The transmission mode of these parasites can be vertical, horizontal or both [24]. Various microsporidian species commonly infecting invertebrates can severely impair host fitness, thereby shaping host population size and dynamics [26–30]. Amphipod-infecting species can compromise their host populations either by i) high virulence causing death of their hosts, ii) a shift in host sex ratio through feminization of the population, iii) impairing growth and/or behaviour, or iv) altering the tolerance to pollutants like heavy metals [15, 27, 31–39]. Previous genetic studies revealed a high diversity of microsporidians in amphipods [35, 40], even in a single population of the indigenous host *G. pulex* in the Ruhr Metropolis [41].

Conclusively, the environmental heterogeneity together with the presence of several indigenous and non-indigenous amphipod species and their microsporidians in the Ruhr Metropolis provides an ideal research platform to study the biological consequences of species invasions into freshwater ecosystems. The aim of the present study was thus

i) to characterize the amphipod and microsporidian assemblages in the study region, and
ii) to investigate whether new host-parasite associations occur in recently invaded freshwater assemblages. This important baseline data will be needed to better understand the process of recolonization of ecologically improved and newly built urban freshwater habitats. Our results may further be used as a reference for future studies investigating the occurrence and prevalence of microsporidians introduced by non-indigenous amphipods (e.g. parasite spillover [5]) or of indigenous microsporidians adapting to new host species (i.e. parasite spillback [20]). Molecular identification via DNA barcoding was performed to identify even juvenile host species and to allow for the assessment of microsporidian infections.

Specifically, we addressed the following hypotheses:

i) Due to the structural heterogeneity and geographical location of the freshwater systems studied, we expect to identify a great diversity of indigenous and non-indigenous host species. However, as amphipod species are relatively well documented for NRW, we do not expect to find cryptic host species. Conversely, and because microsporidian species diversity has only been partially addressed, we expect to find new microsporidian species and new records for the region.
ii) Co-introduced microsporidian species will infect indigenous amphipods in assemblages where they co-occur with non-indigenous hosts (parasite spillover). Also, non-indigenous host species will become infected with indigenous microsporidians (which might lead to parasite spillback). The characterization of indigenous microsporidians (compared to non-indigenous species) can be performed in freshwater regions, which are not yet directly accessible for non-indigenous amphipods. Therefore, the occurrence of a microsporidian lineage in a non-invaded community (e.g. tributaries of the River Emscher separated by polluted sections from the Rhine) characterizes it as being ‘indigenous’.

## Methods

### Sampling

**Fig. 1:**
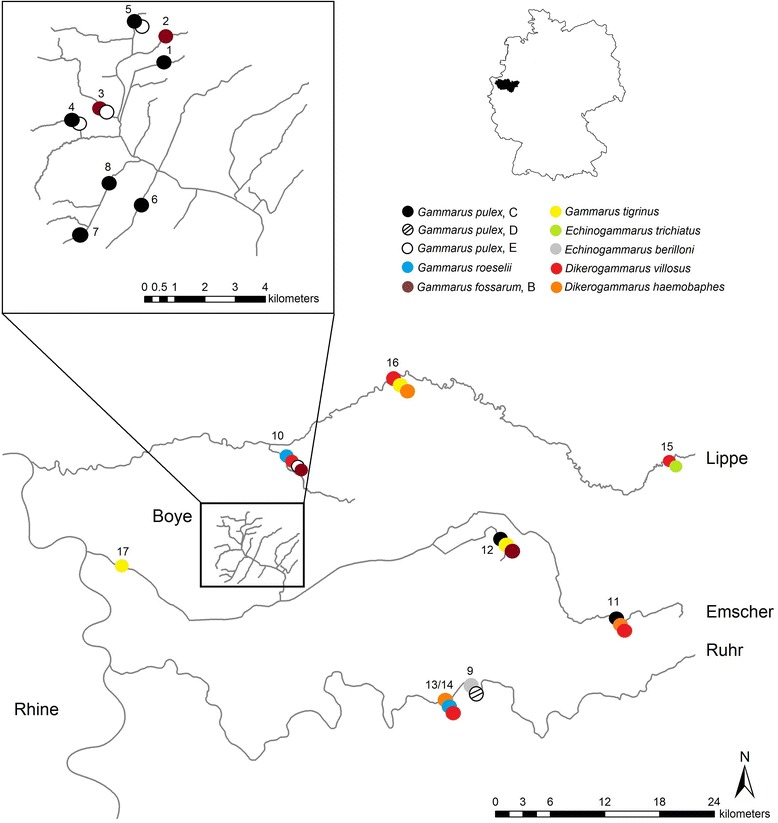
Map showing sampling sites and amphipod lineages/species found. Numbers indicate sampling locations (see also Additional file 1). The Boye-catchment (not influenced by non-indigenous species) is shown in detail Top right: Overview map indicating the position of the study area in the western part of Germany

In total, 17 sampling sites were selected to cover a range of larger and smaller streams as well as more lentic dammed up sites (Fig. 1, see Additional file 1) with a high diversity of indigenous and non-indigenous amphipod species (data from local authorities: Emschergenossenschaft/Lippeverband and [13]) representative for the Ruhr Metropolis. Some of these water bodies were in a near natural state (e.g. Boye tributaries), while others are/were used as sewage channels since the beginning of the 20th century and have been partly restored starting in the 1990s. Hand net samples were taken between October 20th and 29th 2014. Fine sediments and smaller stones were sieved. If larger stones were present, they were turned to dislodge amphipods. Only specimens from Kemnader See and Mühlengraben were collected earlier (17/08/2014). Amphipods were immediately fixed in 96 % ethanol and identified morphologically according to the taxonomic keys of Eggers & Martens [42, 43].

### DNA isolation

If available, 15 specimens of each morphologically identified amphipod species per site were used for molecular analyses. Four to six pereopods of each specimen were used for DNA isolation. For detection of microsporidians, further DNA was extracted from the remaining body tissue. Samples were only analysed for microsporidians if at least five specimens of a single amphipod species were obtained from a single site.

DNA isolation was performed following a modified salt precipitation protocol after Sunnucks & Hales [44]: To each sample, 2 mL TNES Buffer (for amphipod bodies) or 600 μL (for amphipod legs), both containing 0.25 mg/mL proteinase K solution, were added. Samples were incubated overnight at 37 °C. Subsequently, 600 μL of the solution was processed further by adding 170 μL of 5 M NaCl, vortexing and centrifuging for 5 min at 20,000 × g. The supernatant was transferred into a new reaction tube and centrifuged again. The pellet was discarded again and 800 μL of 99 % ice-cold ethanol was added to the supernatant and mixed by repeated inverting. The solution was centrifuged at 20,000 × g for 15 min at 4 °C. The supernatant was discarded, 180 μL 70 % ice-cold ethanol was added for washing. After 15 min centrifugation at 20,000 × g at 4 °C, the ethanol was discarded and the pellet air-dried. The DNA pellet was re-suspended in 50–150 μL TE buffer depending on pellet size.

### PCR and sequencing

For amphipods, the standard animal barcoding locus CO1 was amplified using the degenerated primer pair LCO1490-JJ (5′-CHACWAAYCATAAAGATATYGG-3′) and HCO2198-JJ (5′-AWACTTCVGGRTGVCCAAARAATCA-3′) of Astrin & Stüben [45]. Each PCR reaction mix (total volume of 12.5 μL) contained 1 μL template DNA, 0.2 mM dNTPs, 1x PCR buffer, 0.5 μM of each primer, 0.025 U/μL Hotmaster *Taq*-polymerase (5 PRIME GmbH) and made up to a final volume of 12.5 μL with PCR grade water. PCR cycle conditions were as follows: initial denaturation for 2 min at 94 °C, followed by 36 cycles of 40 s at 94 °C (denaturation), 40 s at 52.5 °C (annealing) and 2 min at 65 °C (elongation), and a final elongation step for 8 min at 65 °C.

Microsporidians were detected using universal microsporidian primers V1 [46]/1342R [47] (Table 1) targeting the small subunit rDNA (SSU rDNA). PCR products were purified (JETQUICK PCR Product Purification Spin Kit, Fa. Genomed), if clear single bands were visible, and were sequenced directly (GATC-Biotech) using primer V1. According to the sequences obtained, species-specific primers were designed for the microsporidian lineages found (Table 1). The remaining samples that could not be sequenced directly, or where sequencing quality was low, were tested with these primers to identify the parasite species. For each primer pair, two randomly selected PCR products were sequenced to assure primer specificity. All SSU rDNA-PCR reactions contained 10 μL of 2× Phire Green PCR Buffer, 0.2 mM dNTP-mix (New England Biolabs), 0.5 μM of each primer, 0.4 μL Phire Green Hot Start II DNA Polymerase (Thermo Scientific) and 1 μL of DNA. PCR grade water was added to 20 μL. PCR conditions for all primers are shown in Table 1.

**Table 1:** Universal and specific primers designed to detect microsporidian species

Primer	Sequence (5′–3′)	Annealing Temperature	Specificity
V1	CAC CAG GTT GAT TCT GCC TGA C	62 °C	universal (Zhu *et al.* 1993, McClymont *et al.* 2005)
1342R	ACG GGC GGT GTG TAC AAA GAA CAG		
Dict11 F	CGA CGT AAA CCT TTT GGT GCA R	60 °C	*Dictyocoela duebenum/ D. muelleri* (Gr/GpC)
Dict11 R	TYT CTT CCG CAA TAC TAA AAA ATT AAT AC		
Dict14 F	GGG CGA TTT ATT TGT TCT CCT GT	58 °C	*Dictyocoela duebenum/ D. muelleri* (Gf/GpE)
Dict14 R	GAT TTC TCT TCC GCA ATA CCA AAT YG		
Dict16/17 F	ATT GAT TAA RGA ACG AGC AGG GTT AG	62 °C	*Dictyocoela duebenum/ D. muelleri* (GpC/Dh)
Dict16/17 R	TCT TCC GCA AYA CMG CCA CA		
Dict15 F	TTT TAA TCG TGG CGT AAA CCA TK	62 °C	*D. berillonum*
Dict15 R	CTC TTC CGC AAT ACA GAA TAC CAT AC		
Mic505 F	CAT CAA CTA ACT TTG GGA AAC TAA G	62 °C	*Microsporidium* sp. 505
Mic505 R	TGG CCT CCC ACA CAT TCC GAG TG		
Mic 515 F	GGC GAT CTA ACC TCG GCA TCG GAT AAC C	62 °C	*Microsporidium* sp. 515
Mic 515 R	TGG CTT CCC ACC CAT TCC GAG C		
Mic 3 F	CAG TAA TGT TGC GAT GAT TTG GTC	58 °C	*Microsporidium* sp. I
Mic 3 R	CAG TAA ATA CTC CAC AGT ATC TTA C		
Mic4 F	TAC GGC TAA GAC GTG GAC	58 °C	*Microsporidium* sp. nov. RR1
Mic4 R	CAA TCC TAT TGC CAT CAT CTG		
Mic6 F	AGG ACC GAC GGC AAA GAA GTC	60 °C	*Microsporidium* sp. nov. RR2
Mic6 R	CAG GAG ATC TCA CCC ATT CAG		
Mic7 F	ACA GTT ATA ATT TAC TCG TAG ATC	58 °C	*Microsporidium* sp. BPAR3
Mic7 R	TAC TCG CAA GCA TGT GCT CA		
Mic18/19 F	ATA GAG GCG GTA GTA ATG AGA CGT A	58 °C	*C. dikerogammari*/ *Microsporidium* sp. G
Mic18/19 R	TTT AAC CAT AAA ATC ACT TCA CTC		

PCR program for all primers was: 98 °C for 3 min, 35 cycles of 98 °C-10 s, [annealing temp. see table]-10 s, 72 °C-15 s (10 s for Dict16/17, 20 s for V1/1342R) and final elongation at 72 °C for 3 min

Prior to sequencing of the CO1 fragment, an ExoI/FastAP purification step was performed. For this purpose, 9 μL of each PCR product were mixed with 1 μL FastAP (1 U/μL) and 0.5 μL ExoI (20 U/μL), both from Thermo Fisher Scientific. The PCR products were enzymatically purified at 37 °C for 25 min and at 85 °C for 15 min. The PCR products were bi-directionally sequenced at GATC Biotech AG using the respective PCR primer pair.

### Sequence editing and alignment

CO1 and SSU rDNA sequences were edited and assembled using Geneious 5.4 [48]. Host and parasite sequences were separately aligned using the Muscle-plugin of Geneious and five iterative runs each. For amphipods, the CO1-sequence of *Crangonyx islandicus* (HM015162) was added to our dataset as an outgroup. For microsporidians, the SSU rDNA-sequence of *Nosema bombycis* (AB093012) was used as an outgroup. The CO1-alignment was manually trimmed at the 5′ and 3′ ends to remove primer sequences. The SSU rDNA-alignment was manually trimmed at the 5′ and 3′ ends to have the majority of SSU rDNA-sequences in the alignment with presence data at both ends.

### Species identification via DNA barcoding

Species identification was performed using BLASTn searches [49] against NCBI GenBank and the Barcode of Life Data System (BOLD) [50]. Additionally, relevant primary literature with available genetic data for amphipods or microsporidians was consulted. Identification was positive, if our query sequence showed at least 98 % sequence identity with one or more reference sequences. A 98 % identity threshold (or 2 % accepted intraspecific variability) was chosen for the identification of amphipods and microsporidian lineages. For the latter, our strategy accounts for potential intragenomic variability present in some microsporidians at the SSU rRNA locus [51], whereas a 2 % threshold for CO1 is far below commonly observed values of interspecific variability in amphipods [52]. For the identification of clades (or potential cryptic species) within *G. pulex* the CO1-alignment of Lagrue *et al.* [53] was used. For visualization of the different host and parasite lineages, Neighbor-Joining trees were calculated with the program MEGA6 [54] under the pairwise-deletion option for the CO1- and SSU rDNA—alignment, respectively. Node support was calculated by 1000 bootstrap replicates. Trees were rooted with the respective outgroup sequence. Outgroups were omitted for the final visualization of trees.

## Results

### Host species identification

**Fig. 2:**
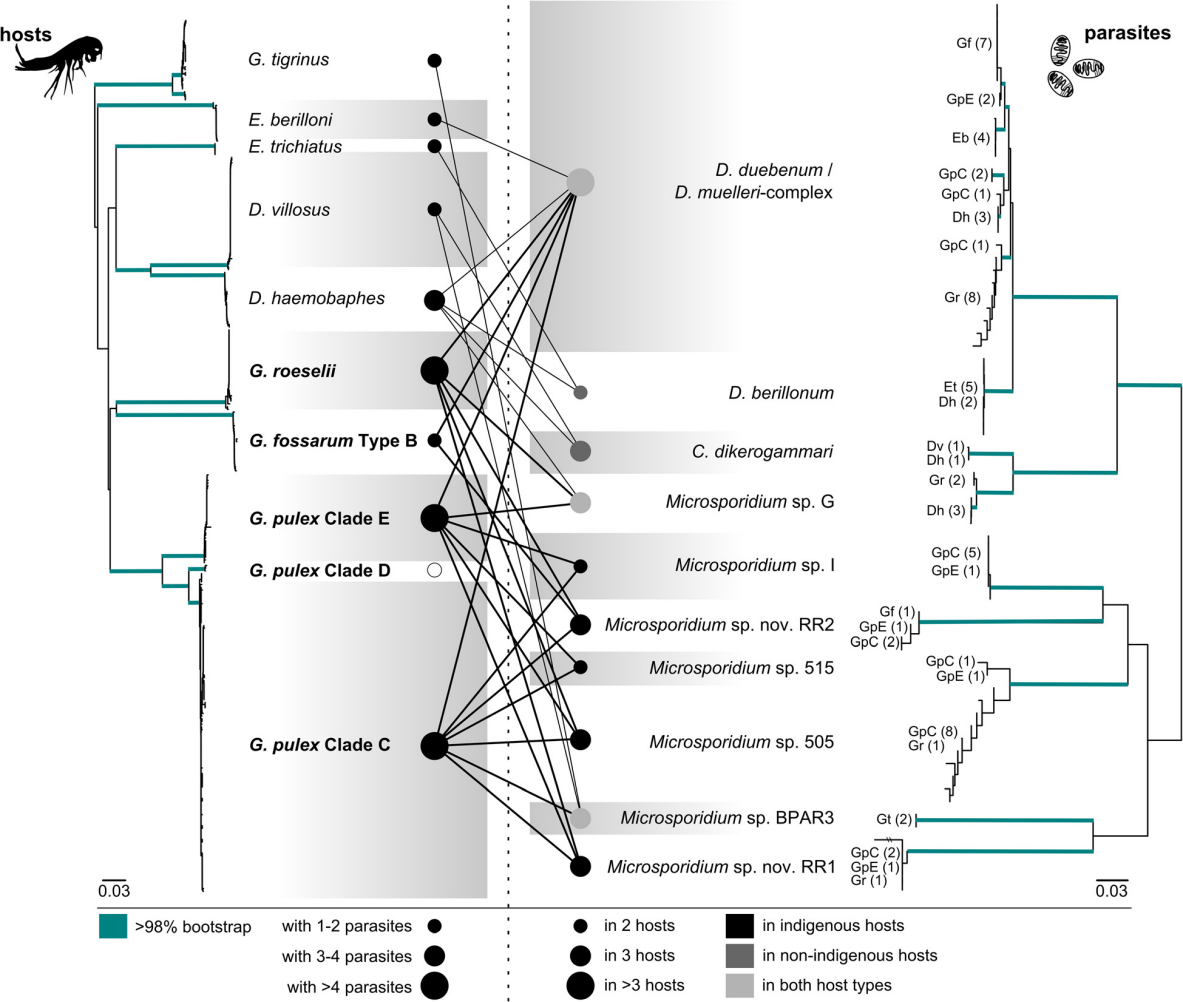
Phylogenetic trees showing the host-parasite relationships of species identified in the present study. Lines indicate which microsporidians were found in the respective host lineage/species. Black lines: microsporidium found in indigenous hosts; grey lines: microsporidium found in non-indigenous hosts. Open circle: not tested for mircosporidians

Our host CO1-dataset (658 bp final alignment length) comprises 319 specimens belonging to eight amphipod species, three indigenous and five non-indigenous, respectively (Table 2, Figs. 1 and 2). Among the former, three clades of *G. pulex* could be distinguished and were treated as separate entities as proposed by Lagrue *et al.* [53]. Two of them (clade C and D) have been previously identified by Lagrue *et al.* [53], whereas the third *G. pulex* clade (here referred to as clade E) was detected e.g. by Hou *et al*. [55]. The single *G. fossarum* species observed can be assigned to *G. fossarum* type B, which is a common amphipod species in Central to Western Europe [56]. *G. pulex* clade D was not tested for parasites, as only three individuals were available. An overview of host haplotypes and estimates of genetic and haplotype diversity can be found in Additional file 2 and Additional file 3, respectively. Genetic diversity of non-indigenous amphipod species was generally low, with *G. tigrinus* being a noticeable exception. This species comprised two genetically distinct lineages (Fig. 2). Furthermore, it showed the highest nucleotide diversity of all species (0.85 %) and the highest haplotype diversity (0.545) as well as the largest number of haplotypes (6) of the non-indigenous species (Additional file 2). All other non-indigenous species harboured a single or only a few haplotypes, potentially indicating a single geographical origin of immigration.

**Table 2:** Identification of host and parasite genetic lineages

Host lineages	Reference
*Gammarus pulex* clade C	Lagrue *et al.* 2014
*Gammarus pulex* clade D	Lagrue *et al*. 2014
*Gammarus pulex* clade E	own designation following Lagrue *et al.* 2014
*Gammarus fossarum* type B	Weiss *et al*. 2014
*Gammarus roeselii*	Hou *et al.* 2011, BOLD
*Gammarus tigrinus*	Costa *et al.* 2009, BOLD
*Echinogammarus berilloni*	Hou *et al.* 2014
*Echinogammarus trichiatus*	Cristescu & Hebert 2005^a^, Arundell *et al.* 2014, BOLD
*Dikerogammarus haemobaphes*	Cristescu & Hebert 2005^a^, BOLD
*Dikerogammarus villosus*	Cristescu & Hebert 2005^a^, Rewicz *et al.* 2015, BOLD
Parasite lineages	
*Dictyocoela duebenum*/*muelleri*- complex	Terry *et al.* 2004, Haine *et al*. 2004, Wilkinson *et al.* 2011
*Dictyocoela berillonum*	Terry *et al.* 2004, Wilkinson *et al*. 2011
*Cucumispora dikerogammari*	Wattier *et al.* 2007, Ovcharenko *et al.* 2010
*Microsporidium* sp. G	Terry *et al*. 2004
*Microsporidium* sp. I	Terry *et al*. 2004, Grabner *et al*. 2014 (=*Microsporidium* M3)
*Microsporidium* sp. 515	Krebes *et al*. 2010, Grabner *et al*. 2014 (=*Microsporidium* M1)
*Microsporidium* sp. 505	Krebes *et al.* 2010, Grabner *et al*. 2014 (=*Microsporidium* M2)
*Microsporidium* BPAR3	Arundell *et al.* 2014
*Microsporidium sp. nov. RR1	own designation
*Microsporidium sp. nov. RR2	own designation

*BOLD* Barcode of Life Database (www.barcodinglife.org). *RR Ruhr-Region

^a^Cristescu ME, Hebert PD. The “Crustacean Seas” an evolutionary perspective on the Ponto-Caspian peracarids. Can J Fish Aquat Sci 2005;62:505–17

### Parasite species identification

The SSU rDNA alignments included sequences of 71 specimens and had a length of 1189 bp. Ten different microsporidian lineages were identified (Table 2, Fig. 2). Eight of them could be assigned to known species of the genus *Dictyocoela*, *Cucumispora dikerogammari* or several species that had been genetically detected in previous studies but lack a formal species description (all classified as *Microsporidium* spp., a dummy group for so far unclassified microsporidian species) (Table 2). Two microsporidian isolates had no close match to any database entry (max. 89 % sequence similarity) and were provisionally treated as *Microsporidium* sp. nov. RR1 and *Microsporidium* sp. nov. RR2 (RR for Ruhr-Region, the broader geographical area of detection).

Due to only minor differences between sequences of *C. dikerogammari* and the closely related *Microsporidium* sp. G as well as the different varieties of the *Dictyocoela duebenum-*/*D. muelleri*- complex, we could not separate all microsporidian isolates from our PCR assays. To assess the prevalence of *C. dikerogammari*, the important co-introduced parasite of *D. villosus*, we sequenced all isolates positive with the Mic 18/19 primers. An overview of haplotypes is given in Additional file 4.

### Host-parasite relationships and prevalences

The most common microsporidians by far belonged to the *D. duebenum-*/*D. muelleri-* complex and were found in both indigenous and non-indigenous hosts (six hosts in total). This complex was subdivided into at least five clades, which seem to be restricted to certain host species or clades. Hosts grouped into a) mostly *G. fossarum* and also *G. pulex* E; b) *E. berilloni*; c) *G. pule*x C; d) *D. haemobaphes*; e) mostly *G. roeselii* and *G. pulex* C (Fig. 2). One specimen of *D. haemobaphes* was simultaneously infected by microsporidia of the *D. duebenum-*/*D. muelleri-* complex and *D. berillonum.*

The highest parasite diversity was found in the indigenous *G. pulex* clades C and E (both with seven parasite species), while *G. fossarum* harbours only two microsporidian species. *Microsporidium* sp. G only occurs in *G. pulex* clade E, not in the most frequent clade C. Overall prevalence in the indigenous amphipods ranged between 50 %–73 % (Table 3).

**Table 3:** Prevalence of microsporidians [%] in each host species and lineage

Host species/clade	no. of host individuals/sites	total pre-valence	*Micro-sporidium* sp. 505	*Micro-sporidium* sp. 515	*Micro-sporidium* sp. I	*Micro-sporidium* sp. nov. RR2	*Micro-sporidium* sp. nov. RR1	*Micro-sporidium* sp. BPAR3	*C. dikero-gammari*	*Micro-sporidium* sp. G	*D. due-benum/D. muelleri*	*D. berill-onum*	undefined	no. of parasite species
*G. pulex* total	149/13	50.34	**20.81**	6.71	4.70	4.70	2.01	2.01	0.00	0.67	4.03	0.00	8.72	8
*G. pulex* clade C	117/9	61.07	**20.51**	8.55	5.13	4.27	1.70	2.56	0.00	0.00	3.42	0.00	7.70	7
*G. pulex* clade E	32/4	50.00	**21.88**	0.00	3.13	6.25	3.13	0.00	0.00	3.13	6.25	0.00	12.50	6
*G. roeselii*	30/2	50.00	6.67	0.00	0.00	3.33	3.33	0.00	0.00	6.67	**26.67**	0.00	3.33	5
*G. fossarum* type B	22/3	72.72	0.00	0.00	0.00	13.64	0.00	0.00	0.00	0.00	**54.55**	0.00	9.10	2
*D. haemobaphes*	21/4	66.67	0.00	0.00	0.00	0.00	0.00	0.00	4.76	23.81	**38.10**	23.81	0.00	4
*D. villosus*	40/5	10.00	0.00	0.00	0.00	0.00	0.00	2.50	2.50	2.50	0.00	0.00	**5.00**	3
*E. berilloni*	15/1	26.67	0.00	0.00	0.00	0.00	0.00	0.00	0.00	0.00	**26.67**	0.00	0.00	1
*E. trichiatus*	5/1	100.00	0.00	0.00	0.00	0.00	0.00	0.00	0.00	0.00	0.00	**100.00**	0.00	1
*G. tigrinus*	28/2	14.29	0.00	0.00	0.00	0.00	0.00	**14.29**	0.00	0.00	0.00	0.00	0.00	1

prevalences of single species might not add up to 100 in all cases as mixed infection occurred; undefined: no result in specific PCR; the most dominant microsporidian species per host species/clade are highlighted in bold

Parasite diversity in the non-indigenous species was generally lower, with a maximum of four species in *D. haemobaphes*. Among all hosts in our study overall prevalence was lowest in the invader *D. villosus* (10 %). In contrast, 67 % of all *D. haemobaphes* individuals were infected. The highest prevalence (100 %) could be found in *E. trichiatus* (Table 3), however, only five individuals were studied from a single site. The non-indigenous parasite *C. dikerogammari* was detected only twice, occurring in *D. villosus* and *D. haemobaphes*.

**Fig. 3:**
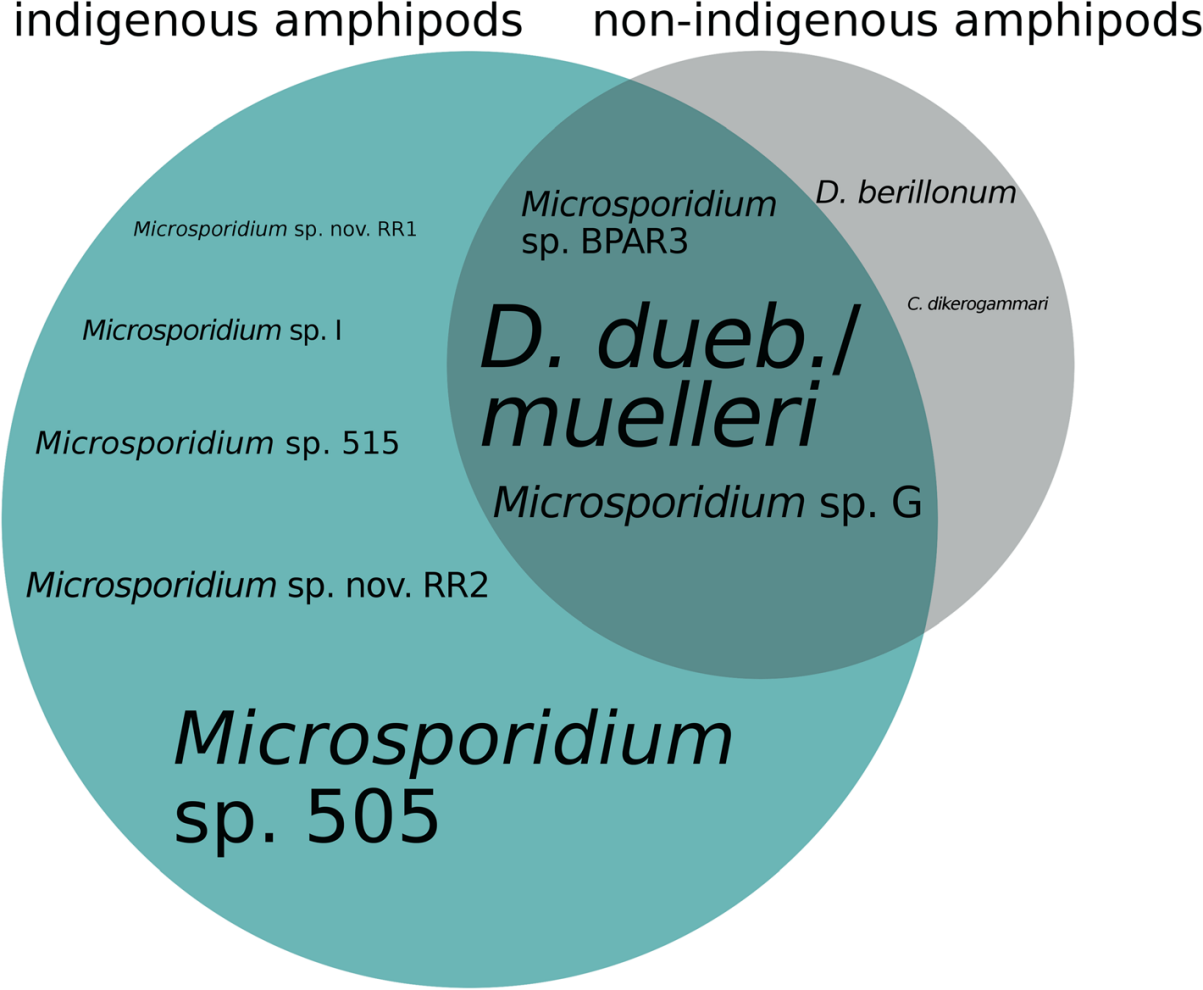
Microsporidians infecting indigenous (green circle) or non-indigenous amphipods (grey circle), as well as microsporidians found in both (overlap area). Size of the circles represents microsporidian prevalence in indigenous /non-indigenous species and font size of microsporidian labels approximately represents the overall prevalence of the respective species

All microsporidians occurred in at least two different host species or clades (Fig. 2), but generally one dominant host species was observed (shown in bold in Table 3). Hence, high prevalence may be generally attributed to infections with two (*Microsporidium* sp. nov. RR2 and *D. duebenum-* /*D. muelleri*- complex in *G. fossarum*; prevalence 14 % and 55 %, respectively), or only one microsporidian species (*D. berillonum* in *E. trichiatus*, prevalence 100 %). Five microsporidian species were only present in native host assemblages of the Boye while only *D. berillonum* was exclusively detected in the non-indigenous amphipods *D. haemobaphes* and *E. trichiatus. Microsporidium* sp. 505 showed the highest prevalence among indigenous hosts, predominantly in *G. pulex* clades C and E. Three microsporidian species (*D. duebenum- */*D. muelleri*- complex, *Microsporidium* sp. BPAR3, *Microsporidium* sp. G) co-occurred in indigenous and non-indigenous amphipods (Figs. 2 and 3).

### Parasite diversity in native and invaded populations

Seven genetically distinct microsporidians were detected in indigenous and so far non-invaded populations of the Boye catchment (Table 4). These microsporidian species may be thus classified as ‘indigenous’ parasites. All of them except *Microsporidium* sp. I were additionally found in invaded populations. In contrast, *C. dikerogammari*, *Microsporidium* sp. G and *D. berillonum* were only present in areas that had already been colonized by non-indigenous species and may be classified as ‘non-indigenous’ parasites. The invaded population from site 10 (Rapphoffs Mühlenbach), a tributary to the River Lippe, had the highest overall host (*n* = 4) and parasite species richness (*n* = 6). Four different microsporidian species were observed at site 13 (Kemnader Lake). In the indigenous populations, host species richness reached a maximum of two (three localities) and parasite richness of five (at site 4, Schöttelbach). At five sites non-indigenous amphipods co-occurred with indigenous species. Although infections were detected at those sites in both indigenous and non-indigenous species, no shared microsporidian species were observed.

**Table 4:** Occurrence data of microsporidian species in native and invaded amphipod populations

Microsporidian species	Native populations (Boye catchment)								Invaded populations (Rivers Emscher, Lippe, Ruhr)								
Site number	1	2	3	4	5	6	7	8	9	10	11	12	13	14	15	16	17
*Dictyocoela duebenum* / *D. muelleri*- complex		x		x	x				x	x	x	x	x				
*Dictyocoela berillonum*	-	-	-	-	-	-	-	-					x		x		
*Cucumispora dikerogammari*	-	-	-	-	-	-	-	-		x						x	
*Microsporidium* sp. G	-	-	-	-	-	-	-	-			x	x	x				
*Microsporidium* sp. 505	x			x	x		x	x		x	x						
*Microsporidium* sp. 515				x	x						x						
*Microsporidium* sp. BPAR3							x	x				x	x			x	
*Microsporidium* sp. nov. RR1	x	x	x	x						x							
*Microsporidium* sp. nov. RR2		x	x							x		x					
*Microsporidium* sp. I			x	x			x	x	-	-	-	-	-	-	-	-	-

Numbers 1–17 refer to the localities sampled. See Additional file 1 for explanation. Dashes indicate microsporidians that were either not found in native or invaded populations

## Discussion

### Diversity of host and parasite species

The first aim of this study was to investigate the diversity of amphipods and their microsporidian parasites in the ecologically heterogeneous freshwater habitats of the Ruhr Metropolis (NRW, Germany). Eight amphipod species were detected of which three are indigenous and five non-indigenous to the region. Thereby, DNA barcoding allowed an unambiguous species identification in all cases even when morphological traits were ambiguous, e.g. in collected juveniles or when delimiting certain individuals of *G. pulex* and *G. fossarum*. All five non-indigenous amphipod species were already recorded for the Ruhr Metropolis, but we have added new occurrence data. High genetic diversity and the separation in two lineages was observed for the non-indigenous species *G. tigrinus.* The indigenous amphipod species *G. pulex* demonstrates a large genetic diversity and consisted of three distinct clades (or potential species) that had been reported in earlier studies, e.g. from France and the Netherlands [53, 55, 57], but not from Germany so far. *G. fossarum* specimens all belonged to type B, which is in agreement with phylogeographic expectations (see [53, 56, 58]).

For microsporidians, DNA barcoding is the only way to assess species diversity, as diagnostic morphological characters applicable for a large scale screening of host individuals are missing. So far, the SSU rDNA marker is consistently sequenced to genetically characterize microsporidians (e.g. [35, 40]), although it was suggested that rDNA is probably not the most suitable marker for microsporidians due to the occurrence of dispersed gene copies within the genome [51]. Nevertheless, our results show a consistent and interpretable pattern of microsporidian sequences, e.g. within a single host species or in comparison to published data from different locations. We hence argue that this marker provides sufficiently conclusive information to differentiate microsporidian species and clades, keeping in mind that the interpretation of results has to be done accurately. Previous studies have already revealed a high diversity of microsporidians in a variety of European amphipods [35, 40], sometimes even in a single host population [33, 41, 59]. Freshwater amphipod populations analysed so far mainly originated from France, Ireland and the UK. When comparing the published data with our findings of ten different microsporidian species (seven indigenous, three non-indigenous) for the Ruhr Metropolis, we find a large overlap of microsporidian species in our study region with the aforementioned regions. Thus, a wide geographic distribution and low host-specificity of some microsporidian species is obvious.

The two (presumed) non-indigenous parasites detected in the present study (see argumentation below) contribute to the total microsporidian diversity, which was higher at invaded sites with nine microsporidian species compared to the non-invaded sites where seven species occurred. However, when taking into account the number of host species available (including the different *G. pulex* clades), an average of 0.9 microsporidian species per host lineage were found at the invaded sites, while 2.3 microsporidians per host lineage were detected at the sites not influenced by non-indigenous amphipods. This shows that microsporidian diversity relative to the number of potential hosts is in fact higher in uninvaded regions. It should be mentioned that not all host species were represented equally in the total sample. *G. pulex* clade C was most dominant concerning sites (9) and number of individuals (117) and harboured the highest number of microsporidian species (7). Nevertheless, we believe that this sampling bias does not have a strong effect on the results as also underrepresented species such as *G. roeselii* (30 specimens, 2 sites) were parasitized by a large number of microsporidians (5).

### Host-parasite interactions, parasite spillover and spillback

The second aim was to assess (potentially new) host-parasite interactions, especially among non-indigenous hosts and indigenous parasites or vice versa. Thereby, we intended to reveal possible cases of parasite spillover or parasite spillback that might affect indigenous host populations.

Among the ten microsporidians found in the present study, the *D. duebenum- */*D. muelleri-* complex seems to be the most common in European amphipods. It was found in a variety of host species and shows the widest distribution among all amphipod-infecting microsporidians investigated [35, 38, 40, 59–63]. This species complex was also most abundant in the present study and our data can add *G. pulex* clade C, E and *G. fossarum* type B as new hosts to this parasite group. By assigning *D. duebenum* and *D. muelleri* as a single species complex, we follow Wilkinson *et al.* [62] who did not find clear support for *D. muelleri* as a separate species in their haplotype analysis of European-wide isolates. However, the same authors demonstrated that several host-specific genetic lineages exist within this complex, which can be confirmed with our data. Furthermore, Wilkinson *et al.* [62] discussed the possibility of horizontal transmissions for this parasite in certain cases. Few, if any, horizontal transmissions are expected in vertically transmitted microsporidians [64, 65], which otherwise might allow parasite clones to switch to new host species. On the contrary, a prevailing vertical mode of transmission will ‘lock’ the parasite to the host species, allowing the formation of host species-associated lineages, as it was observed in the present study and by Wilkinson *et al.* [62]. Interestingly, the *D. duebenum-* /*D. muelleri*- complex was not detected in the non-indigenous amphipods *D. villosus*, *E. trichiatus* and *G. tigrinus,* which are supposed to be susceptible to this parasite [35, 60, 63]. If the aforementioned hypothesis is true, no host switch event has yet occurred that would have allowed the establishment of *D. duebenum- */*D. muelleri*- complex in the local populations of these amphipods.

In the present study, all microsporidians were detected in at least two different host species. The strongest host-specificity of the investigated microsporidians can most likely be attributed to *Microsporidium* sp. I, which has been exclusively isolated from *G. pulex* (see also [35, 40, 41]. However, as it occurs in two of the distinct *G. pulex* clades, which probably represent different species, no final statement about its host specificity can currently be made. Nevertheless, a certain degree of host specificity seems to exist as microsporidians present in multiple host species were usually found in one host species in considerably higher prevalence.

Among the ten microsporidian species found in the present study, only *C. dikerogammari* and *D. berillonum* can be unambiguously designated as non-indigenous parasites to the Ruhr Metropolis as both were exclusively detected in non-indigenous amphipods (*C. dikerogammari* in *D. villosus* and *D. haemobaphes*, *D. berillonum* in *D. haemobaphes* and *E. trichiatus*; *D. berillonum* also previously detected in *D. villosus* from the Netherlands [63]). *Cucumispora dikerogammari* was described as a typical, co-introduced parasite of *D. villosus* [60]. Horizontal transmission of this microsporidian to other host species seems possible, but only if the prevalence in the ‘source’ *D. villosus*-population is high [21]. *C. dikerogammari* was detected only twice in our study, infecting a single specimen of *D. villosus* and *D. haemobaphes*, respectively. Therefore, we assume that the current risk of *C. dikerogammari* spreading to indigenous amphipod species in the Ruhr Metropolis is low. However, *C. dikerogammari* can usually be detected in prevalences of up to 50 % [19, 60] and the question remains which processes lead to the observed low prevalence pattern in our study region. When testing various populations of *D. villosus* along the River Rhine, Wattier *et al.* [60] did not find any indication of parasite release from *C. dikerogammari*. In their study, prevalence was found to be low at more recently colonized sites, but it increased quickly within a few years. In contrast, Bojko *et al.* [66] and Arundell *et al*. [63] did not detect *C. dikerogammari* in UK populations of *D. villosus*. These authors explained their findings with bottleneck effects for the host and parasite release upon the invasion of *D. villosus* in the UK. This apparent discrepancy between prevalences might be illustrated by the more continuous invasion of *D. villosus* within Europe (along rivers) and contrasts with the punctual, accidental anthropogenic introductions of smaller groups of individuals in the UK. The virtual lack of *C. dikerogammari* (and other microsporidian) infections in *D. villosus* in the Ruhr Metropolis might be explained by rare, punctual introductions, leading to bottleneck events in founder populations similar to what has been proposed for *D. villosus* in the UK. *D. villosus* was first observed in 1996 in the River Lippe, where it has been isolated at one site far upstream from the mouth of the river (data of the Federal Environmental Agency and the Lippeverband). At this time, no populations were detected further downstream, which can be seen as support for a punctual introduction event with subsequent parasite release. Furthermore, this observation may indicate a dominant role of ship transportation as a potential mode of passive dispersal for this species.

An alternative explanation for the low prevalence’s observed in *D. villosus* may come from the frequent co-occurrence with its congener *D. haemobaphes*. It is known that *C. dikerogammari* not only impairs survival, but also reduces the competitive strength of its amphipod host [67]. Both closely related species might be strong competitors and competition might select for *D. villosus* individuals with low susceptibility to the parasite, thereby eliminating the parasite from the host population. This hypothesis is supported by the study of Ebert *et al*. [68], who modeled the impact of microparasites on host populations. Their results imply that either the host or the parasite will be eliminated from the population if the parasite is highly virulent, as it is the case for *C. dikerogammari*. The only *D. villosus* specimen infected with *C. dikerogammari* was found at a site where *D. haemobaphes* was absent, which might be seen as further evidence for our argumentation. On the other hand, *D. haemobaphes* itself was highly infected with several microsporidians, mostly by lineages of the *D. duebenum- */*D. muelleri*- complex that are supposed to be non-virulent vertical transmitters [35]. An apparent lack of effects of a vertically transmitted microsporidian species on the competitive potential and the colonization success of an amphipod host was shown for *D. berillonum* in invasive populations of *D. haemobaphes* in the UK [69] and can be generally expected for non-virulent vertically transmitted parasites [17, 32, 70]. The North American invader *Crangonyx pseudogracilis* may serve as another example: Although a genetic bottleneck effect was detected for host populations in Europe, they have formed established populations together with two co-introduced, vertically transmitted microsporidian species [71].

The other non-indigenous microsporidium, *D. berillonum*, was found in *E. trichiatus* from the Lippe and in *D. haemobaphes* from “Kemnader Lake”, a more lentic reservoir lake of the River Ruhr. However, this parasite species was not present in the *E. berilloni* population located in the River Ruhr upstream of the site “Kemnader Lake”. *E. berilloni* would be a suitable host for *D. berilloni* [35], but an apparently exclusive mode of vertical transmission prevents a host switch in this case.

*Microsporidium* sp. G may be another non-indigenous species in the Ruhr Metropolis as we found the parasite only at sites influenced by non-indigenous amphipods. It was detected in the non-indigenous *D. haemobaphes*, as well as in the indigenous *G. pulex* and *G. roeselii*. To our knowledge, a similar microsporidian isolate (98 % sequence identity) has only been described once from the host *Gammarus chevreuxi* in the UK [35]. Additional records for this parasite will allow a more conclusive interpretation of its natural distribution range.

*Microsporidium* sp. BPAR3 was present at invaded and two adjacent uninvaded sites infecting indigenous (*G. pulex*, *G. roeselii*) as well as non-indigenous amphipods (*D. villosus*, *G. tigrinus*). Theoretically, this wide host tolerance would be an ideal prerequisite for an invader. *Microsporidium* sp. BPAR3 was initially detected in the amphipod *Dorogostaiskia parasitica* from lake Baikal (unpublished, NCBI accession no. FJ756100; 98 % sequence similarity). Lake Baikal is rather isolated with respect to its amphipod fauna [72], but still parasites from endemic host species are observed throughout European freshwaters. More recently, Arundell *et al.* [63] isolated a microsporidian from *E. trichiatus* collected in the Netherlands that genetically matched closely our two sequenced isolates from *G. tigrinus* (99.6 % sequence identity). These findings are further examples for the wide geographical distribution of some microsporidian species. However, an introduction of an amphipod from Lake Baikal to central Europe happened only in the case of the intentional transfer of *Gmelinoides fasciatus* to the Baltic Sea drainage, but so far this species has not reached German inland waters [73]. Hence, an introduction of Baikalian parasites to the Ruhr Metropolis seems rather unlikely. On the contrary, *Microsporidium* sp. BPAR3 could be regarded as a widely distributed parasite with unknown origin, occurring in Western Europe and/to Lake Baikal in Central Asia.

Passive transportation of freshwater species (including parasites), e.g. by birds as a potential mechanism causing large or patchy distribution patterns was shown for various aquatic organisms including amphipods, which can attach to legs or feathers [74]. Most likely, such mechanism explain the isolated presence of *Microsporidium* sp. BPAR3 on a larger geographical scale. On a more regional scale, passive transportation (by birds) may be an important mechanism for non-indigenous amphipods in the Ruhr Metropolis occurring at sites not accessible by other mechanisms, for example in the unpolluted upstream section of the River Emscher where *D. villosus* and *D. haemobaphes* populations are found. The nearby Lake Phoenix (an artificial urban freshwater lake) is attractive for various species of waterfowl and introduction of amphipods and their parasites (here *Microsporidium* sp. G) has likely originated from the Lippe or Ruhr catchments. Additionally, amphipods and their parasites may have been anthropogenically co-introduced with the new lake flora.

Finally, we revealed two microsporidian lineages, *Microsporidium* sp. nov. RR1 and RR2 that to our knowledge have not been detected in previous genetic studies. Most likely, they are local natives as they also occur in the uninvaded streams of the Boye-system. In general, the findings of local microsporidian species compared to more widely distributed species highlights the remarkable differences in the potential of these parasites to spread from one to the next host population or to follow the dispersal routes of their host species.

## Conclusion

By using DNA barcoding we revealed a high diversity of microsporidian parasites in their sometimes ‘cryptic’ amphipod hosts, thereby revealing two new microsporidian isolates (*Microsporidium* sp. nov. RR1 and RR2) and at least two non-indigenous parasites that have been co-introduced with their non-indigenous amphipod hosts. In agreement with earlier studies, our data highlights the broad distribution of some microsporidians in Europe (and beyond) in disconnected host populations without clear indication for a recent invasion. We found more parasite species in invaded amphipod assemblages, but when corrected for the number of available host lineages, parasite diversity was higher in uninvaded than in invaded sites.

Our study suggests that the risk for the native amphipod fauna in the Ruhr Metropolis due to non-indigenous microsporidians is low as no indication for parasite spillover or spillback was found. Nevertheless, this might change in the future, e.g. if *C. dikerogammari* can adapt to indigenous amphipod species or if new invaders import new parasites. This risk is obvious as with the continuous improvement of the water quality in the local rivers, especially the River Emscher for which three non-indigenous species have already been reported (*G. tigrinus*, *D. villosus*, *D. haemobaphes*), new direct pathways for invaders and their parasites will be opened in the future.

## Additional files

Additional file 1:
**Sampling locations; Names and GPS coordinates of sampling sites.** (PDF 180 kb) Additional file 2:
**Genetic data of host amphipod species.** Given are the number of specimens (N), number of haplotypes (H), haplotype diversity (Hd) and genetic diversity (π); sd = standard deviation. (PDF 262 kb) Additional file 3:
**Haplotypes of amphipod species and accession numbers.** (XLSX 12 kb) Additional file 4:
**Haplotypes of microsporidian isolates and accession numbers.** (XLSX 12 kb)

## References

[1] Hulme PE (2009). Trade, transport and trouble: managing invasive species pathways in an era of globalization. J Appl Ecol.

[2] Banks NC, Paini DR, Bayliss KL, Hodda M (2015). The role of global trade and transport network topology in the human-mediated dispersal of alien species. Ecol Lett.

[3] Elton CS. The Ecology of Invasions by Animals and Plants. Chicago: University of Chicago Press; 2000.

[4] Sures B, Simberloff D, Rejmánek M (2011). Parasites of animals. Encyclopedia of Biological Invasions.

[5] Dunn AM, Hatcher MJ (2015). Parasites and biological invasions: parallels, interactions, and control. Trends Parasitol.

[6] McKinney ML (2006). Urbanization as a major cause of biotic homogenization. Biol Conserv.

[7] Kowarik I (2011). Novel urban ecosystems, biodiversity, and conservation. Environ Pollut.

[8] Navel S, Mermillod-Blondin F, Montuelle B, Chauvet E, Simon L, Piscart C (2010). Interactions between fauna and sediment control the breakdown of plant matter in river sediments. Fresh Biol.

[9] Piscart C, Mermillod-Blondin F, Maazouzi C, Merigoux S, Marmonier P (2011). Potential impact of invasive amphipods on leaf litter recycling in aquatic ecosystems. Biol Invasions.

[10] Eiseler B (2010). Taxonomie für die Praxis. Bestimmungshilfen - Makrozoobenthos (1) LANUV-Arbeitsblatt 14.

[11] Bij de Vaate A, Jazdzewski K, Ketelaars HAM, Gollasch S, van der Velde G (2002). Geographical patterns in range extension of Ponto-Caspian macroinvertebrate species in Europe. Can J Fish Aquat Sci.

[12] Rewicz T, Wattier R, Grabowski M, Rigaud T, Bacela-Spychalska K (2015). Out of the Black Sea: Phylogeography of the invasive Killer Shrimp *Dikerogammarus villosus* across Europe. PLoS One.

[13] Winking C, Lorenz AW, Sures B, Hering D (2014). Recolonisation patterns of benthic invertebrates: A field investigation of restored former sewage channels. Freshw Biol.

[14] Dick JTA, Platvoet D, Kelly DW (2002). Predatory impact of the freshwater invader *Dikerogammarus villosus* (Crustacea: Amphipoda). Can J Fish Aquat Sci.

[15] MacNeil C, Dick JTA, Hatcher MJ, Dunn AM (2003). Differential drift and parasitism in invading and native *Gammarus* spp. (Crustacea: Amphipoda). Ecography.

[16] MacNeil C, Dick JTA, Hatcher MJ, Terry RS, Smith JE, Dunn AM (2003). Parasite-mediated predation between native and invasive amphipods. Proc Biol Sci.

[17] Prenter J, Macneil C, Dick JTA, Dunn AM (2004). Roles of parasites in animal invasions. Trends Ecol Evol.

[18] Dunn AM (2009). Parasites and biological invasions. Adv Parasitol.

[19] Ovcharenko MO, Bacela K, Wilkinson T, Ironside JE, Rigaud T, Wattier R (2010). *Cucumispora dikerogammari* n. gen. (Fungi: Microsporidia) infecting the invasive amphipod *Dikerogammarus villosus*: a potential emerging disease in European rivers. Parasitology.

[20] Kelly DW, Paterson RA, Townsend CR, Poulin R, Tompkins DM (2009). Parasite spillback: a neglected concept in invasion ecology?. Ecology.

[21] Bacela-Spychalska K, Wattier RA, Genton C, Rigaud T (2012). Microsporidian disease of the invasive amphipod *Dikerogammarus villosus* and the potential for its transfer to local invertebrate fauna. Biol Invasions.

[22] Britton JR (2013). Introduced parasites in food webs: new species, shifting structures?. Trends Ecol Evol.

[23] Williams BA, Hirt RP, Lucocq JM, Embley TM (2002). A mitochondrial remnant in the microsporidian *Trachipleistophora hominis*. Nature.

[24] Smith JE (2009). The ecology and evolution of microsporidian parasites. Parasitology.

[25] Stentiford GD, Feist SW, Stone DM, Bateman KS, Dunn AM (2013). Microsporidia: diverse, dynamic, and emergent pathogens in aquatic systems. Trends Parasitol.

[26] Kohler SL, Wiley MJ (1992). Parasite-induced collapse of populations of a dominant grazer in Michigan streams. Oikos.

[27] Dunn A, Smith J (2001). Microsporidian life cycles and diversity: the relationship between virulence and transmission. Microbes Infect.

[28] Kohler SL, Hoiland WK (2001). Population regulation in an aquatic insect: the role of disease. Ecology.

[29] Decaestecker E, Declerck S, De Meester L, Ebert D (2005). Ecological implications of parasites in natural *Daphnia* populations. Oecologia.

[30] Zbinden M, Haag CR, Ebert D (2008). Experimental evolution of field populations of *Daphnia magna* in response to parasite treatment. J Evol Biol.

[31] Bandi C, Dunn A, Hurst G, Rigaud T (2001). Inherited microorganisms, sex-specific virulence and reproductive parasitism. Trends Parasitol.

[32] Dunn AM, Terry RS, Smith JE (2001). Transovarial transmission in the microsporidia. Adv Parasitol.

[33] Ironside JE, Smith JE, Hatcher MJ, Sharpe RG, Rollinson D, Dunn AM (2003). Two species of feminizing microsporidian parasite coexist in populations of *Gammarus duebeni*. J Evol Biol.

[34] Kelly A, Hatcher MJ, Dunn AM (2003). The impact of a vertically transmitted microsporidian, *Nosema granulosis* on the fitness of its *Gammarus duebeni* host under stressful environmental conditions. Parasitology.

[35] Terry RS, Smith JE, Sharpe RG, Rigaud T, Littlewood DTJ, Ironside JE (2004). Widespread vertical transmission and associated host sex-ratio distortion within the eukaryotic phylum Microspora. Proc Biol Sci.

[36] Haine ER, Motreuil S, Rigaud T (2007). Infection by a vertically-transmitted microsporidian parasite is associated with a female-biased sex ratio and survival advantage in the amphipod *Gammarus roeseli*. Parasitology.

[37] Mautner SI, Cook KA, Forbes MR, McCurdy DG, Dunn AM (2007). Evidence for sex ratio distortion by a new microsporidian parasite of a Corophiid amphipod. Parasitology.

[38] Gismondi E, Rigaud T, Beisel J-N, Cossu-Leguille C (2012). Microsporidia parasites disrupt the responses to cadmium exposure in a gammarid. Environ Pollut.

[39] Gismondi E, Rigaud T, Beisel J-N, Cossu-Leguille C (2012). Effect of multiple parasitic infections on the tolerance to pollutant contamination. PLoS One.

[40] Krebes L, Blank M, Frankowski J, Bastrop R (2010). Molecular characterisation of the Microsporidia of the amphipod *Gammarus duebeni* across its natural range revealed hidden diversity, wide-ranging prevalence and potential for co-evolution. Infect Genet Evol.

[41] Grabner DS, Schertzinger G, Sures B (2014). Effect of multiple microsporidian infections and temperature stress on the heat shock protein 70 (hsp70) response of the amphipod *Gammarus pulex*. Parasit Vectors.

[42] Eggers TH, Martens A (2001). A key to the freshwater Amphipoda (Crustacea) of Germany. Lauterbornia.

[43] Eggers TO, Martens A (2004). Additions and Corrections to “A key to the freshwater Amphipoda (Crustacea) of Germany”. Lauterbornia.

[44] Sunnucks P, Hales DF (1996). Numerous transposed sequences of mitochondrial cytochrome oxidase I-II in aphids of the genus *Sitobion* (Hemiptera: Aphididae). Mol Biol Evol.

[45] Astrin JJ, Stüben PE (2008). Phylogeny in cryptic weevils: molecules, morphology and new genera of western Palaearctic Cryptorhynchinae (Coleoptera: Curculionidae). Invertebr Syst.

[46] Zhu X, Wittner M, Tanowitz HB, Kotler D, Cali A, Weiss LM (1993). Small subunit rRNA sequence of *Enterocytozoon bieneusi* and its potential diagnostic role with use of the polymerase chain reaction. J Infect Dis.

[47] McClymont EH, Dunn AM, Terry RS, Rollinson D, Littlewood DTJ, Smith JE. Molecular data suggest that microsporidian parasites in freshwater snailsare diverse. Int J Parasitol. 2005;35:1071–8. 10.1016/j.ijpara.2005.05.008 16023122

[48] Kearse M, Moir R, Wilson A, Stones-Havas S, Cheung M, Sturrock S (2012). Geneious Basic: an integrated and extendable desktop software platform for the organization and analysis of sequence data. Bioinformatics.

[49] Altschul SF, Gish W, Miller W, Myers EW, Lipman DJ (1990). Basic local alignment search tool. J Mol Biol.

[50] Ratnasingham S, Hebert PD (2007). BOLD: The Barcode of Life Data System (http://www.barcodinglife.org). Mol Ecol Notes.

[51] Ironside JE (2013). Diversity and recombination of dispersed ribosomal DNA and protein coding genes in microsporidia. PLoS One.

[52] Costa FO, Henzler CM, Lunt DH, Whiteley NM, Rock J (2009). Probing marine *Gammarus* (Amphipoda) taxonomy with DNA barcodes. Syst Biodivers.

[53] Lagrue C, Wattier R, Galipaud M, Gauthey Z, Rullmann J-P, Dubreuil C (2014). Confrontation of cryptic diversity and mate discrimination within *Gammarus pulex* and *Gammarus fossarum* species complexes. Freshw Biol.

[54] Tamura K, Stecher G, Peterson D, Filipski A, Kumar S (2013). MEGA6: Molecular Evolutionary Genetics Analysis version 6.0. Mol Biol Evol.

[55] Hou Z, Sket B, Fiser C, Li S (2011). Eocene habitat shift from saline to freshwater promoted Tethyan amphipod diversification. Proc Natl Acad Sci U S A.

[56] Weiss M, Macher JN, Seefeldt MA, Leese F (2014). Molecular evidence for further overlooked species within the *Gammarus fossarum* complex (Crustacea: Amphipoda). Hydrobiologia.

[57] Hou Z, Fu J, Li S (2007). A molecular phylogeny of the genus *Gammarus* (Crustacea: Amphipoda) based on mitochondrial and nuclear gene sequences. Mol Phylogenet Evol.

[58] Westram AM, Jokela J, Baumgartner C, Keller I (2011). Spatial distribution of cryptic species diversity in European freshwater Amphipods (*Gammarus fossarum*) as revealed by pyrosequencing. PLoS One.

[59] Haine ER, Brondani E, Hume KD, Perrot-Minnot M-J, Gaillard M, Rigaud T (2004). Coexistence of three microsporidia parasites in populations of the freshwater amphipod *Gammarus roeseli*: evidence for vertical transmission and positive effect on reproduction. Int J Parasitol.

[60] Wattier RA, Haine ER, Beguet J, Martin G, Bollache L, Muskó IB (2007). No genetic bottleneck or associated microparasite loss in invasive populations of a freshwater amphipod. Oikos.

[61] Yang G, Short S, Kille P, Ford AT (2010). Microsporidia infections in the amphipod, *Echinogammarus marinus* (Leach): suggestions of varying causal mechanisms to intersexuality. Mar Biol.

[62] Wilkinson TJ, Rock J, Whiteley NM, Ovcharenko MO, Ironside JE (2011). Genetic diversity of the feminising microsporidian parasite *Dictyocoela*: new insights into host-specificity, sex and phylogeography. Int J Parasitol.

[63] Arundell K, Dunn A, Alexander J, Shearman R, Archer N, Ironside JE (2014). Enemy release and genetic founder effects in invasive killer shrimp populations of Great Britain. Biol Invasions.

[64] Ironside JE, Dunn AM, Rollinson D, Smith JE (2003). Association with host mitochondrial haplotypes suggests that feminizing microsporidia lack horizontal transmission. J Evol Biol.

[65] Krebes L, Zeidler L, Frankowski J, Bastrop R (2013). (Cryptic) sex in the microsporidian *Nosema granulosis* - Evidence from parasite rDNA and host mitochondrial DNA. Infect Genet Evol.

[66] Bojko J, Stebbing PD, Bateman KS, Meatyard JE, Bacela-Spychalska K, Dunn AM (2013). Baseline histopathological survey of a recently invading island population of “killer shrimp”, *Dikerogammarus villosus*. Dis Aquat Organ.

[67] Bacela-Spychalska K, Rigaud T, Wattier RA (2014). A co-invasive microsporidian parasite that reduces the predatory behaviour of its host *Dikerogammarus villosus* (Crustacea, Amphipoda). Parasitology.

[68] Ebert D, Lipsitch M, Mangin K (2000). The effect of parasites on host population density and extinction: experimental epidemiology with *Daphnia* and six microparasites. Am Nat.

[69] Green Etxabe A, Short S, Flood T, Johns T, Ford A (2014). Pronounced and prevalent intersexuality does not impede the “Demon Shrimp” invasion. PeerJ.

[70] Drake JM (2003). The paradox of the parasites: implications for biological invasion. Proc Biol Sci.

[71] Slothouber Galbreath JGM, Smith JE, Becnel JJ, Butlin RK, Dunn AM (2010). Reduction in post-invasion genetic diversity in *Crangonyx pseudogracilis* (Amphipoda: Crustacea): a genetic bottleneck or the work of hitchhiking vertically transmitted microparasites?. Biol Invasions.

[72] Väinölä R, Witt JDS, Grabowski M, Bradbury JH, Jazdzewski K, Sket B (2008). Global diversity of amphipods (Amphipoda; Crustacea) in freshwater. Hydrobiologia.

[73] Panov VE, Berezina NA, Leppäkoski E, Gollasch S, Olenin S (2002). Invasion History, Biology and Impacts of the Baikalian Amphipod *Gmelinoides fasciatus*. Invasive Aquatic Species of Europe. Distribution, Impacts and Management.

[74] Figuerola J, Green AJ (2002). Dispersal of aquatic organisms by waterbirds: A review of past research and priorities for future studies. Freshw Biol.

