# Supplementary material for: Invaders, natives and their enemies: distribution patterns of amphipods and their microsporidian parasites in the Ruhr Metropolis, Germany
Source: Parasit Vectors. 2015 Aug 13;8:419. doi: 10.1186/s13071-015-1036-6 (PMC4534018; doi:10.1186/s13071-015-1036-6)
Supplement: Additional file 1: — Sampling locations; Names and GPS coordinates of sampling sites. (PDF 180 kb) [file 13071_2015_1036_MOESM1_ESM.pdf]

Additional file 1. Sampling locations

Site number	Site name	Coordinates (lat.; long.)
1	Quälingsbach	51.5856151; 6.9540089
2	Brabecker Mühlenbach	51.5934367; 6.9537975
3	Boye	51.5690727; 6.9287250
4	Schöttelbach	51.5639119; 6.9117179
5	Bornemannsbach	51.5983583; 6.9386889
6	Kirchschemmsbach	51.5437030; 6.9405765
7	Vorthbach 42	51.5358507; 6.9139819
8	Vorthbach 43	51.5435291; 6.9205793
9	Ruhr upstream Kemnader Lake	51.4208960; 7.2966850
10	Rapphoffs Mühlenbach close to mouth into Lippe	51.6643410; 6.9762790
11	Emscher near Lake Phönix	51.4867500; 7.5230420
12	Deininghauser Brook	51.5812470; 7.3192820
13	Kemnader Lake	51.4229100; 7.2679480
14	Mühlengraben, branch of Kemnader Lake	51.4271230; 7.2859530
15	Lippe near Werne	51.6692020; 7.6992860
16	Lippe near Haltern	51.7328950; 7.1774890
17	Emscher close to mouth into Rhine	51.5566070; 6.7213380
