# Supplementary material for: Invaders, natives and their enemies: distribution patterns of amphipods and their microsporidian parasites in the Ruhr Metropolis, Germany
Source: Parasit Vectors. 2015 Aug 13;8:419. doi: 10.1186/s13071-015-1036-6 (PMC4534018; doi:10.1186/s13071-015-1036-6)
Supplement: Additional file 2: — Genetic data of host amphipod species. Given are the number of specimens (N), number of haplotypes (H), haplotype diversity (Hd) and genetic diversity (π); sd = standard deviation. (PDF 262 kb) [file 13071_2015_1036_MOESM2_ESM.pdf]

Additional file 2. Genetic data of host amphipod species. Given are the number of specimens (N), number of haplotypes (H), haplotype diversity (Hd) and genetic diversity (π); sd = standard deviation.

**Indigenous host species**	N	H	Hd	sd	π	sd
G. pulex clade C	117	22	0.868	0.019	0.0035	0.0002
G. pulex clade D	3	3	1.000	0.272	0.0020	0.0007
G. pulex clade E	33	6	0.333	0.105	0.0006	0.0002
G. fossarum type B	23	3	0.170	0.102	0.0003	0.0002
G. roeselii	30	5	0.361	0.109	0.0011	0.0004
**Non-indigenous host species**						
D. villosus	42	3	0.138	0.071	0.0003	0.0001
D. haemobaphes	21	2	0.524	0.036	0.0008	0.0001
G. tigrinus	30	6	0.545	0.101	0.0085	0.0027
E. berilloni	15	3	0.257	0.020	0.0006	0.0004
E. trichiatus	5	1	0.000	0.000	0.0000	0.0000
